# Registered Nurse Employment and Earnings in Long-Term Care

**DOI:** 10.1093/geroni/igaf122.1836

**Published:** 2025-12-31

**Authors:** Joanne Spetz, Timothy Bates

**Affiliations:** University of California, San Francisco, San Francisco, California, United States; University of California, San Francisco, San Francisco, California, United States

## Abstract

Objectives This study identifies RN characteristics associated with employment in LTC and factors associated with wage differences between RNs working in LTC versus other settings. Methods We analyzed data from the 2022 US National Sample Survey of Registered Nurses. We calculated descriptive statistics and conducted statistical tests of differences in the characteristics of RNs employed in LTC versus other settings and of their hourly wages. We then estimated a logistic regression to identify demographic, human capital, and employment characteristics that predict LTC employment. We estimated a linear regression with the logarithm of wage as the dependent variable to measure wage differences between LTC and other RNs, holding other characteristics constant. Results Our study sample had 14,216 RNs, of whom 1,334 were employed in LTC settings. RNs employed in LTC were older than RNs in other settings, and the racial-ethnic composition was similar between the groups. LTC-employed RNs were more likely to have completed an associate degree versus a bachelor’s degree, and to have been educated outside the US. RNs in LTC earned an average of 9.5% less than other RNs. Among RNs in LTC settings, there were significantly lower hourly wages for those with management and education job titles, compared with staff nurse titles, and no differences by age group, race/ethnicity, education, or gender. Conclusions Because RNs in LTC earn lower wages than RNs in other settings, policies to minimize pay inequities are needed to support the RN workforce providing LTC services.

